# Evaluation of the CRISPR/Cas9 Genetic Constructs in Efficient Disruption of Porcine Genes for Xenotransplantation Purposes Along with an Assessment of the Off-Target Mutation Formation

**DOI:** 10.3390/genes11060713

**Published:** 2020-06-26

**Authors:** Natalia Ryczek, Magdalena Hryhorowicz, Daniel Lipiński, Joanna Zeyland, Ryszard Słomski

**Affiliations:** 1 Department of Biochemistry and Biotechnology, Poznan University of Life Sciences, Dojazd 11, 60-632 Poznań, Poland; magdalena.hryhorowicz@gmail.com (M.H.); lipinskidaniel71@gmail.com (D.L.); jzeyland@gmail.com (J.Z.); 2 Institute of Human Genetics, Polish Academy of Sciences, Strzeszyńska 32, 60-479 Poznań, Poland; ryszard.slomski@up.poznan.pl

**Keywords:** xenoantigen, coagulation system dysregulation, CRISPR/Cas9 system, genome modifications, non-homologous DNA ends joining (NHEJ), TIDE analysis, off-target

## Abstract

The increasing life expectancy of humans has led to an increase in the number of patients with chronic diseases and organ failure. However, the imbalance between the supply and the demand for human organs is a serious problem in modern transplantology. One of many solutions to overcome this problem is the use of xenotransplantation. The domestic pig (*Sus scrofa domestica*) is currently considered as the most suitable for human organ procurement. However, there are discrepancies between pigs and humans that lead to the creation of immunological barriers preventing the direct xenograft. The introduction of appropriate modifications to the pig genome to prevent xenograft rejection is crucial in xenotransplantation studies. In this study, porcine *GGTA1*, *CMAH*, *β4GalNT2*, *vWF*, *ASGR1* genes were selected to introduce genetic modifications. The evaluation of three selected gRNAs within each gene was obtained, which enabled the selection of the best site for efficient introduction of changes. Modifications were examined after nucleofection of porcine primary kidney fibroblasts with CRISPR/Cas9 system genetic constructs, followed by the tracking of indels by decomposition (TIDE) analysis. In addition, off-target analysis was carried out for selected best gRNAs using the TIDE tool, which is new in the research conducted so far and shows the utility of this tool in these studies.

## 1. Introduction

The increasing life expectancy of humans has led to an increase in the number of patients with chronic diseases and organ failure. Organ transplantation is an effective approach in the treatment of the end-stage organ failure. However, the imbalance between the supply and the demand for human organs is a serious problem in modern transplantology. In view of the above, it is assumed that alternative approaches would reduce or eliminate this problem. One of many solutions is the use of xenotransplantation [1,2].

Xenotransplantation is any procedure that involves the transplantation, implantation, or infusion of a recipient (in this case a human) with zoonotic cells, tissues, or organs. In addition, therapies using human body fluids, tissues, organs, or cells that have had ex vivo contact with animal organs, tissues, or cells are also subject to this term [3]. The domestic pig (*S. s. domestica*) is currently considered as the most suitable species for human organ procurement. The reasons for choosing the pig as a donor animal include its relatively large litter size and short puberty, the size and physiological similarity of its organs to human organs, and the low risk of xenozoonosis transmission [4]. However, there are discrepancies between pigs and humans that lead to the creation of immunological barriers preventing the direct xenograft. These differences cause xenograft rejection [5]. The introduction of appropriate modifications into the porcine genome to prevent xenograft rejection is crucial in xenotransplantation studies. There are three main types of xenograft rejection consecutively—hyperacute rejection (HAR), acute humoral xenograft rejection (AHXR), and acute cellular rejection (ACR) [6,7,8]. In addition, dysregulation of the recipient’s coagulation system is a barrier in xenotransplantation, which appears in parallel with HAR, AXHR, and ACR [9,10]. Accordingly, this study focuses on preventing HAR and coagulation dysregulation in xenotransplantation.

HAR is a process that occurs within a few minutes to several hours after xenotransplantation. HAR is a type of humoral rejection mediated by IgM antibodies naturally occurring in the recipient. The association of recipient antibodies with epitopes present on the porcine endothelial cells activates the complement system [11]. To date, three epitopes have been described that constitute a barrier to xenotransplantation and are responsible for HAR. Galactose-α1,3-galactose (α-Gal) is the major xenoantigen involved in xenograft hyperacute rejection. This epitope is synthesized by α-1,3-galactosyltransferase encoded by the porcine *GGTA1* gene [11,12]. The second significant epitope is Neu5Gc, which is formed in an enzymatic reaction involving cytidine monophospho-N-acetylneuraminic acid hydroxylase encoded by the porcine *CMAH* gene [13,14]. β-1,4 N-acetylgalactosaminyltransferase 2 encoded by the porcine *β4GalNT2* gene is involved in the synthesis of Sd(a) antigen [15,16,17].

Dysregulation of the recipient’s coagulation system is one of the main barriers in xenotransplantation. It causes the development of thrombotic microangiopathy in xenograft. Features of thrombotic microangiopathy include fibrin deposition and platelet aggregation, which causes thrombosis within the transplant blood vessels and ultimately ischemic damage [18]. With the development of disorders of the coagulation system, systemic consumption coagulopathy is often observed in the recipient, which can lead to his death [19]. There are also factors expressed in specific organs that pose a problem in xenotransplantation. One of them is the von Willebrand factor encoded by the porcine *vWF* gene, which is involved in the pathogenesis of transplant failure in lung xenotransplantation [20,21]. Fatal thrombocytopenia accompanying liver xenotransplantation is another barrier resulting from differences in the coagulation system. Human platelets are bound by asialoglycoprotein receptor (ASGR) encoded by porcine *ASGR1* and *ASGR2* genes. They are expressed in liver sinusoidal endothelial cells (LSEC) [22,23].

The described research focuses on introducing changes within genes that are involved in the immune response that is the biggest barrier in xenotransplantation. Porcine *GGTA1*, *CMAH*, and *β4GalNT2* genes involved in the synthesis of epitopes responsible for the xenograft hyperacute rejection. Additionally, two genes responsible for the synthesis of porcine proteins causing dysregulation of the recipient’s coagulation system have been chosen—the porcine *vWF* gene and porcine *ASGR1* gene.

The use of the CRISPR/Cas9 system for genome modification has led to enormous progress in the field of animal transgenesis [24]. Through projectable short gRNA, it is possible to precisely generate double-strand DNA breaks (DSBs). DSBs of DNA generated by the CRISPR/Cas9 system are repaired by NHEJ or by homologous directed repair (HDR) after delivery of properly designed donor DNA [25,26]. There are, however, some restrictions related to the use of the CRISPR/Cas9 system. One of them is off-target effect [27]. In this study, the usefulness of the TIDE online tool has been confirmed not only to analyze indel-type mutations arising as a result of DSBs repair via NHEJ but also to analyze additional Cas9 hydrolysis sites linked to a specific gRNA—off-target sites.

## 2. Materials and Methods

### 2.1. Selection of Short Oligonucleotides

Short oligonucleotides (gRNA) and PCR primers were selected using the Benchling platform, San Francisco, CA, USA (www.benchling.com). Nucleotides have been added to the selected gRNA sequences at the 5’ and 3’ ends for molecular cloning. The designed oligonucleotides were ordered from the Institute of Biochemistry and Biophysics Polish Academy of Sciences.

### 2.2. Preparation of Genetic Constructions in the CRISPR/Cas9 System

The constructions were prepared using the vector *pSpCas9(BB)-2A-Puro (PX459) V2.0*, which was a gift from Feng Zhang (Addgene plasmid #62988; http://n2t.net/addgene:62988; RRID: Addgene_62988). For each pair of oligonucleotides, a hybridization mixture containing 100 μM F and R gRNA sequences was prepared. The mixtures were incubated 5 min at 95 °C and then incubated at room temperature for 10 min. Plasmid DNA was hydrolyzed with the restriction enzyme *Bbs*I-HF^®^ (New England Biolabs, Ipswich, MA, USA) for 1.5 h at 37 °C. To combine oligonucleotides after hybridization with plasmids in linear form, ligation was performed using T4 DNA Ligase (New England Biolabs, Ipswich, MA, USA). The mixtures were incubated for 18 h at 16 °C, followed by additional hydrolysis with the use of enzyme *Bbs*I-HF^®^ for 30 min at 37 °C. Then *Escherichia coli* bacterial cells were transformed with purified constructions and positive clones were selected. Plasmid DNA isolation was performed using the Plasmid Maxi Kit (Qiagen, Germantown, MD, USA).

### 2.3. Isolation and Culture of Porcine Primary Kidney Cells In Vitro

For the isolation of porcine primary kidney fibroblasts, a 2 × 2 × 2 cm kidney cortical tissue was excised. Then, after tissue fragmentation, enzymatic-mechanical disintegration was carried out using collagenase II (Sigma-Aldrich, Darmstadt, Germany) and a magnetic stirrer. Then the incubation was carried out for 60 min while heating the mixture to 37 °C. The mixture was then filtered through a filter (0.5 mm pore diameter) and washed several times in the culture medium (DMEM (Sigma-Aldrich, Darmstadt, Germany), 20% FBS (Sigma-Aldrich, Darmstadt, Germany), 1% MEM Non-Essential Amino Acid Solution (100×) (Sigma-Aldrich, Darmstadt, Germany)), 1% Antibiotic Antimycotic Solution (100×) (Sigma-Aldrich, Darmstadt, Germany), 1% Sodium pyruvate solution (100 mM) (Sigma-Aldrich, Darmstadt, Germany), 1% L-Glutamine solution (200 mM) (Sigma-Aldrich, Darmstadt, Germany)). In vitro cell culture was carried out under standard aseptic conditions (5% CO_2_, 37 °C).

### 2.4. Nucleofection

Primary porcine kidney fibroblasts were detached from the surface of T-flasks by Accutase^®^ solution (Sigma-Aldrich, Darmstadt, Germany) and counted using a Scepter^TM^ Handheld Automated Cell Counter, version 2.0 (Merck, Darmstadt, Germany). One million cells were used for the transfection. Nucleofection was carried out using the Mouse Embryonic Fibroblast Nucleofector™ Kit 1 (Lonza, Basel, Switzerland) and program T-007 on Amaxa™ Nucleofector™ II device (Lonza, Basel, Switzerland).

### 2.5. Antibiotic Selection

After 24 h since performing nucleofection, the culture medium has been changed to selective culture medium with the addition of puromycin at a concentration of 1 μg/mL. Incubation was then carried out for 48 h, after which the selection medium was removed, and standard medium was added again. The culture was carried out until the expected cell confluency was obtained.

### 2.6. Analysis of Introduced Genetic Modifications

Prior to analysis of the introduced genetic modifications, DNA isolation from modified primary porcine kidney fibroblasts was performed using the DNeasy Blood & Tissue Kit (Qiagen, Germantown, MD, USA). Then PCR reactions were performed using the StartWarm HS-PCR Mix (A&A Biotechnology, Gdynia, Poland) to amplify DNA fragments that include modified *loci* and DNA fragments containing potential off-target sites. The purified PCR products were sequenced in the Sequencing Laboratory of the Faculty of Biology at the Adam Mickiewicz University in Poznan.

### 2.7. Sequencing Analysis Using the TIDE Tool

The presence of modifications at the target locus and off-target sites after nucleofection of primary porcine kidney fibroblasts was identified using the TIDE online tool, version 2.0.1 by The Netherlands Cancer Institute, Amsterdam, Netherlands (https://tide.deskgen.com/).

## 3. Results

### 3.1. Selection of the Potential Modifications Location in Porcine Genome

The first stage of research was to find the best bioinformatically selected gRNA using the Benchling platform. The choice was made based on several guidelines: the localization of the introduced modifications, the values of the on-target score (from 0 to 100—the higher score, the better) and the off-targets score (from 0 to 100—the higher the score, the lower the chance of additional genomic cleavage sites). The localization of the three selected gRNAs for each of the tested *loci* in the porcine genome is shown in Table 1.

### 3.2. Analysis of On-Target Modification Sites

#### 3.2.1. Nucleofection Efficiency

**Figure 1:**
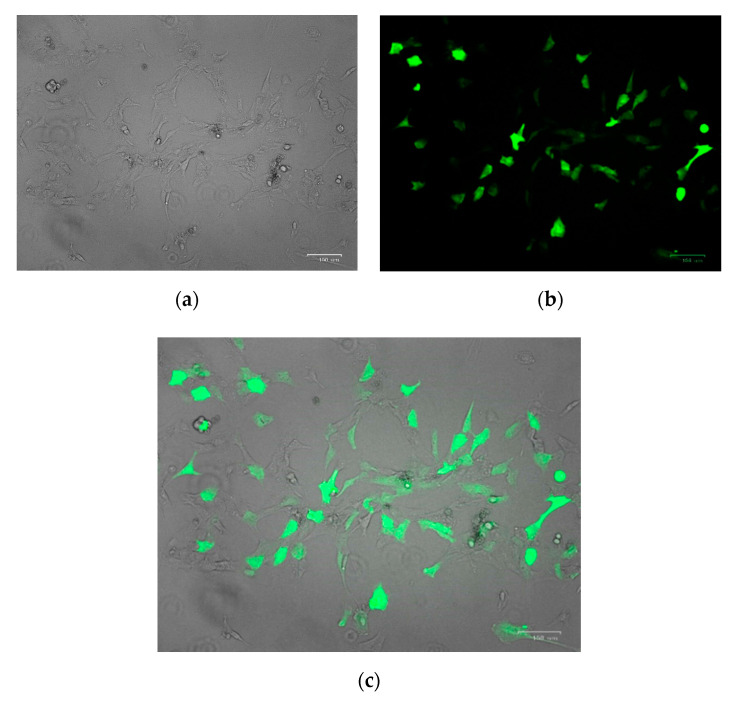
Nucleofection efficiency obtained on porcine primary kidney fibroblasts. Cells visualization was performed using a ZOE Fluorescent Cell Imager 24 h after nucleofection—(**a**) brightfield; (**b**) fluorescence detection lamp; (**c**) merge of view (**a**,**b**). The scale is 100 µm.

To obtain genetically modified primary porcine renal fibroblasts, nucleofections were performed. Transfection efficiency for isolated cells was checked using the *pmaxGFP^TM^ Vector* from the nucleofection kit and visualized using a ZOE Fluorescent Cell Imager (Figure 1). After counting 10,000 cells from multiple views, transfection efficiency was estimated to be around 65–70%.

#### 3.2.2. The Efficiency of Introducing Modifications within the Examined Porcine Genes Using Genetic Constructions Selected as the Best

PCR products obtained on DNA template isolated from modified and unmodified porcine primary kidney fibroblasts were purified using a CleanUp kit (A&A Biotechnology, Gdynia, Poland). Then, sequencing was commissioned, the results of which were analyzed using the TIDE online tool, version 2.0.1. One, best gRNA from the three directed at each of the examined genes was selected.

The gGGTA1 F1/R1 was chosen as the best for the disruption of the porcine *GGTA1* gene. The gGGTA1 F1/R1, when combined with Cas9, enabled on modification of porcine primary kidney fibroblasts with total efficiency of 70.1%. A statistically significant mutation occurring with the highest frequency was a single nucleotide deletion in 51.1% sequences. It was determined that the insertion of one nucleotide occurred at 11.6% (adenosine nucleotide was incorporated in 47.6% sequences).

From the genetic constructs of the CRISPR/Cas9 system directed to the porcine *CMAH* gene, the one containing gCMAH F3/R3 was chosen as the best, as it enabled obtaining 61.2% total efficiency of modification. The most common mutation was the insertion of a single nucleotide, occurring in 57% of the tested sequences. In 93.6% of sequences, the inserted nucleotide was the cytidine.

The next gene tested was porcine *β4GalNT2*. For genetic construction containing gβ4GalNT2 F3/R3, a total modification efficiency of 45.2% was obtained. The most common mutation was a deletion of one nucleotide, which occurred in 28.8% sequences. The second most common mutation was the insertion of a single nucleotide, occurring in 8.4% of cases. Adenosine nucleotide was inserted in 79.8% of sequences.

Another analysis was performed for genetic constructions directed at the porcine *vWF* gene. A total efficiency of 85.1% was obtained for the plasmid containing gvWF F2/R2. The most common deletion of one nucleotide occurred with a frequency of 39.9%. The second most common mutation was the insertion of one nucleotide, which occurred in 34.3% of sequences. An adenosine nucleotide was inserted in 83.6% of sequences.

The last gene tested was porcine *ASGR1*. A total efficiency of 80.5% was obtained for the plasmid containing gASGR1 F3/R3. The most common deletion of one nucleotide occurred in 58.6% of sequences. The second most common mutation was the deletion of two nucleotides.

**Figure 2:**
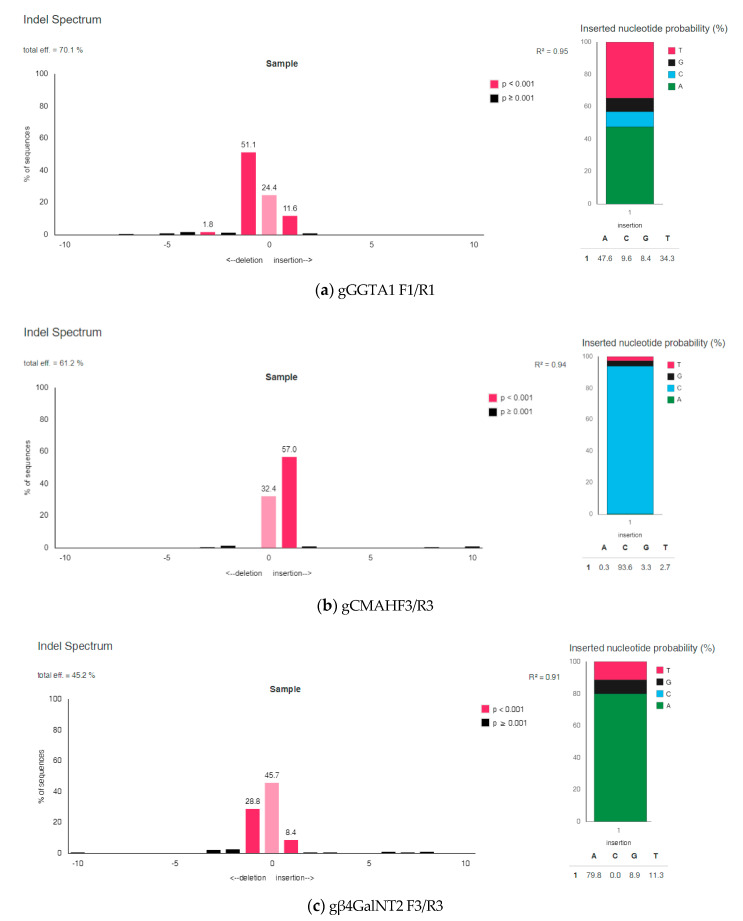

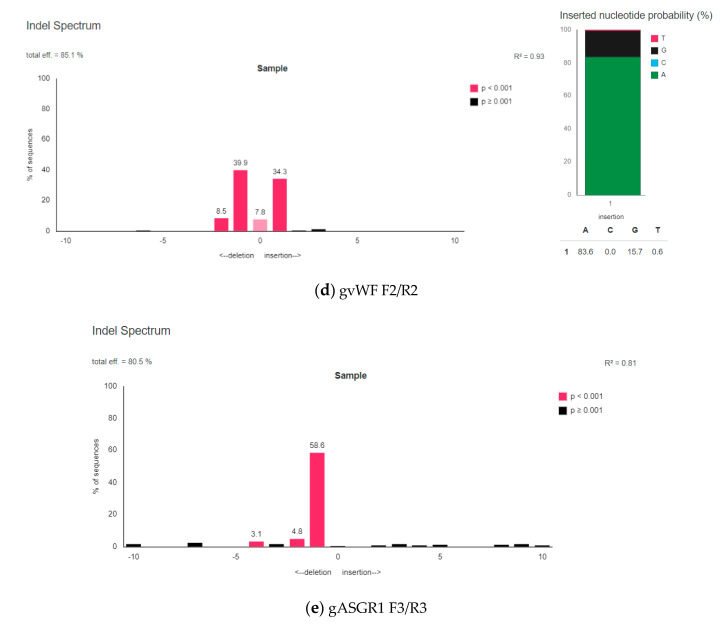
The indel spectrum and inserted nucleotide probability results for the CRISPR/Cas9 genetic constructs containing gRNA chosen as the best for disruption of tested porcine genes. Results obtained after use the plasmids with (**a**) gGGTA1 F1/R1; (**b**) gCMAH F3/R3; (**c**) gβ4GalNT2 F3/R3; (**d**) gvWF F2/R2; (**e**) gASGR1 F3/R3.

Detailed results of indel spectrum and inserted nucleotide probability for all chosen as the best gRNAs are presented in Figure 2.

The indel spectrum and inserted nucleotide probability results, alignment, and decomposition quality controls for the other CRISPR/Cas9 genetic constructs containing gRNA tested for disruption of the porcine genes are present in the Supplement (Figure S1). The alignment and decomposition quality controls for the CRISPR/Cas9 genetic constructs containing gRNA chosen as the best for the disruption of the porcine genes are present in the Supplement (Figure S2).

#### 3.2.3. Comparison of Bioinformatically Predicted DNA Diruption Efficiency Results with Those Obtained in the In Vitro Cultured Cells

The total efficiency results of modifications obtained using the CRISPR/Cas9 system predicted during bioinformatics analysis in silico were compared with those obtained in in vitro cultured porcine primary kidney fibroblasts. Comparison of the results for all tested construction variants is shown in Table 2.

### 3.3. Analysis of the Potential Off-Target Sites in Porcine Genome

#### 3.3.1. Selection of the Potential Off-Target Sites Location

After selecting the best genetic constructs containing gRNAs that mediate in the efficient disruption of studied porcine loci, off-target sites were predicted. For each targeted modification site, a minimum of three loci have been selected that can lead to mutations outside the target locus using the Benchling internet platform. The main criterion considered when choosing the tested off-target sites was the score showing the probability of a DNA break at a given off-target site by the testes construct is equal to or higher than 1.0. In addition, all off-target loci predicted in the coding sequences for selected constructs were checked. The list of tested potential off-target sites is presented in Table 3.

#### 3.3.2. TIDE Analysis of the Chosen Potential Off-Target Sites

**Figure 3:**
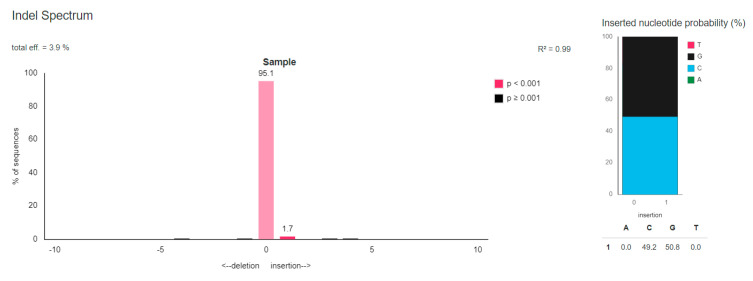
The indel spectrum and inserted nucleotide probability result for the number 1 off-target locus after the use of CRISPR/Cas9 genetic construct containing gGGTA1 F1/R1 chosen as the best for disruption of porcine *GGTA1* gene.

The presence of unwanted changes—the indel mutations, in eight off-target sites from the 19 tested loci was confirmed using the TIDE tool. The off-target sites were confirmed in *loci* numbers 1, 4, 5, 10, 11, 14, 18, and 19. Only in one locus the occurred modification was statistically significant at the level *p* < 0.001 and it was site number 1. It was determined that the off-target site number 1 for the genetic construct of gGGTA1 F1/R1 was cut with a total efficiency of 3.9%. A mutation arising with the statistical significance was the insertion of one nucleotide, which occurred in 1.7% sequences. Guanosine nucleotide was inserted in the 50.8% sequences. The indel spectrum and inserted nucleotide probability for the number 1 off-target locus is presented in Figure 3.

#### 3.3.3. Comparison of Bioinformatically Predicted DNA Diruption Efficiency Results in the Off-Target Sites with Those Obtained in the In Vitro Cultured Cells

The efficiency results of off-target mutation using the CRISPR/Cas9 system obtained during bioinformatics analysis were compared with those obtained in in vitro cultures. The results are summarized in Table 4.

## 4. Discussion

The research presented in this paper shows the importance of the verification of the bioinformatically selected gRNAs in the context of the modifications obtaining efficiency. To compare the efficiency of individual gRNAs in modifications obtaining in cells cultured in vitro, it was necessary to use the third-generation CRISPR/Cas9 system, which enables to carry out antibiotic selection using puromycin after transfection. This process eliminates the impact of the transfection efficiency associated with the chosen method on the efficiency of obtaining modifications via individual gRNAs [28,29,30]. After antibiotic selection, a population of positively transfected cells after nucleofection was examined. In this way, it was possible to determine the efficiency of indel mutation formation at target sites for specific gRNAs. It was shown that the results obtained in the cells prepared in this way slightly coincided with bioinformatics predictions.

Therefore, the selection of gRNA itself, which would mediate the disruption of specific genomic site should be given the great importance. There are many different online tools that enable gRNA design. The limiting factor are the available databases related to the genomes of various organisms. Thus, the selection of the appropriate tool for prediction of Cas9 DNA hydrolysis target sites is based on the chosen research model [31,32].

The results of sequencing of PCR products that were amplified within the target sites of designed gRNAs were analyzed using the TIDE tool. This method makes it easy to determine, based on the results of sequencing, the indel mutation profile that occurs in a population of genetically modified cells using the CRISPR/Cas9 system. The indel detection by amplicon analysis (IDAA) method is also used for this purpose. It is based on three-primer amplicon labeling and detection by capillary electrophoresis [33]. The sensitivity and precision of both methods to determine the insertion-deletion profile of the modification is similar. The choice of method used depends on the researcher’s preferences and available infrastructure [34,35].

Based on the results obtained in this study, the best targeted sites were selected within the genes tested, thanks to which it is possible to obtain modifications efficiently. The assessment of individual gRNAs is very important in the context of obtaining simultaneous multimodification of the porcine genome for xenotransplantation purposes. This is the latest goal of researchers in this field. Research related to this work is part of this trend. Modifications used in the research are aimed at counteracting the two immune barriers existing in xenotransplantation. The knock-out of the porcine *GGTA1*, *CMAH* and *β4GalNT2* genes aims at the elimination of the hyperacute xenograft rejection. Prevention of the coagulation dysregulation can be achieved by the disruption of the porcine *vWF* gene for lung xenotransplantation and knock-out of the porcine *ASGR1* gene for liver xenotransplantation.

An important aspect related to these results is the ethical effect. Preliminary evaluation of bioinformatic data allows to limit the participation of animals in research. This is in line with the Polish recommendations of the National Ethics Committee for Animal Experiments described in Resolution No. 14/2017, as well as with international guidelines and trends.

This study also analyzes the off-target sites generated by Cas9 nuclease in combination with the best gRNAs selected. The formation of additional DNA hydrolysis sites by Cas9 is a common problem observed in CRISPR/Cas9-related studies. Off-target mutations are the biggest threat associated with the use of the new genome editing technology. In total, 19 potential off-target sites were tested for genetic constructs selected during experiments (Figure 3, Figures S3–S7). Off-target mutations were confirmed at eight loci. Only in one of them the changes occurred with statistical significance at the level of *p* < 0.001. This was site No. 1 (for genetic construction containing gGGTA1 F1/R1). Eight potential off-target sites were in the coding sequences of the porcine genome. Experimentally, in genetically modified porcine primary kidney fibroblast cultures, the presence of the off-target mutations in two gene coding sequences was confirmed. The first locus in which off-target mutation has been reviled after the use of the genetic construction with gvWF F2/R2 lies within the porcine *COMT* gene. Another one confirmed off-target site for gASGR1 F3/R3 containing constructs is within the porcine *C1orf210* gene. These loci must be checked after receiving genetically modified animals using obtained using these two genetic constructs.

The obtained results correlate with the research that describe the influence of the non-complementary nucleotides (mismatch nucleotides) position between gRNA and the sequence of the potential off-target site. The efficiency of the off-target mutation formation is higher when incomplementarity occurs in the eighth nucleotide and in the first three nucleotides of the gRNA sequence (5′→3′). For nucleotide in the eighth position (5′→3′) such a relationship was confirmed for two out of eight off-target sites (numbers 1 and 19) for the examined gRNAs in this study. In addition, as many as seven out of eight off-target sites (numbers 1, 4, 5, 10, 11, 18, and 19) had a mismatch of at least one nucleotide in the first, second, or third position of the gRNA sequence (5′→3′) [36]. Interestingly, this study also revealed that in three out of eight confirmed off-target sites, complementarity was found for the seventh position nucleotide. Studies show that the CRISPR/Cas9 system tolerates three to five mismatches in the distal gRNA region from the protospacer adjacent motif (PAM) sequence. In addition, it has been reported that the complementary alignment of the ten nucleotides of the proximal gRNA region from the PAM sequence is sufficient to mediate the Cas9 nuclease DNA hydrolysis [37]. It has also been shown that the occurrence of nucleotide incomplementarity at positions 15, 16, and 17 of the gRNA sequence (5′→3′) to a potential off-target site abolishes CRISPR/Cas9 system activity [38].

The research presented above proved the usefulness of the TIDE online tool for off-target sites checking. There are other, more accurate methods used for this purpose. One of them is DISCOVER-Seq (discovery of in situ Cas off-targets and verification by sequencing), which uses recruitment of the factors involved in DNA repair. To this end, the binding of double-strand break DNA repair protein (MRE11) is tracked. Thanks to this it is possible to check the double-strand DNA breaks in the whole genome [39]. Another method is CIRCLE-seq (circularization for in vitro reporting of cleavage effects by sequencing), it consists of next generation sequencing and leads to the analysis of the presence of off-target sites throughout the genome [40]. Both above methods have many advantages, so it would be worth to additionally perform these analyzes for the best genetic constructions selected in this paper. However, their use is only possible in in vitro cell culture studies and it is not possible to evaluate off-target sites in genetically modified animals. In contrast, the TIDE tool enables quick and cheap recognition of basic off-target sites, and its use can apply to both cellular and model animals research.

New approaches have emerged to reduce the risk of off-target mutations after using the CRISPR/Cas9 system. One of them is the use of new algorithms in bioinformatics tools, thanks to which it is possible to more accurately assess the efficiency of modifications within individual off-target sites [41,42,43,44]. Another approach is to use modified Cas9 with nickase activity. It has been proven that introducing modifications with the CRISPR/Cas9 system modified in such a way reduces the number of unwanted mutations [45,46]. The second approach is to provide Cas9 protein and gRNA in the form of a ribonucleoprotein complex (RNP). It has been shown that this method of delivery of the CRISPR/Cas9 system enabled obtaining the modifications at the target site with high efficiency. The number of the off-target mutations that have arisen has significantly decreased. However, the use of the RNP complex has a major drawback. There is a problem with the number of complexes delivered. Very high concentrations are used because only such concentrations can guarantee that some of them will function in the cell nuclei of genetically modified cell cultures in vitro. High concentration of the RNP complexes may have a cytotoxic effect on some cell lines [47,48,49,50,51]. Accordingly, ribonucleoprotein complexes can only be used in some experiments.

## 5. Conclusions

The research presented above enabled the usefulness assessment of the CRISPR/Cas9 system genetic constructions containing gRNAs to obtain modifications for xenotransplantation purposes. Selected gRNAs, which in combination with Cas9 nuclease enable for the efficient disruption of the studied genes, can be used to obtain genetically modified pigs. To obtain an effect associated with counteracting the immune response in the recipient—the primate animal models or human, all the modifications tested should be present in the porcine genome simultaneously. The genetically modified porcine primary kidney fibroblasts cells nuclei for the SCNT procedure or verified genetic constructs for microinjection of the porcine zygotes can be used to obtain the desired porcine genome modifications. After receiving genetically modified animals, it is necessary to check off-target sites, which presence has been confirmed by the above analyzes.

## Figures and Tables

**Table 1:** Localization of the potential modification *loci* in porcine genome.

Porcine Genome Locus	gRNA	Exon	Chromosome Localization ^1^
*GGTA1*	gGGTA1 F1/R1	Exon 8	Chromosome 1, c261513705-261513686 (NC_010443.5)
	gGGTA1 F2/R2	Exon 8	Chromosome 1, c261513541-261513522 (NC_010443.5)
	gGGTA1 F3/R3	Exon 8	Chromosome 1, c261513764-261513745 (NC_010443.5)
*CMAH*	gCMAH F1/R1	Exon 6	Chromosome 7, c19902027-19902008 (NC_010449.5)
	gCMAH F2/R2	Exon 3	Chromosome 7, c19917616-19917597 (NC_010449.5)
	gCMAH F3/R3	Exon 5	Chromosome 7, c19903792-19903773 (NC_010449.5)
*β4GalNT2*	gβ4GalNT2 F1/R1	Exon 2	Chromosome 12, c25388178-25388159 (NC_010454.4)
	gβ4GalNT2 F2/R2	Exon 3	Chromosome 12, c25386323-25386304 (NC_010454.4)
	gβ4GalNT2 F3/R3	Exon 6	Chromosome 12, c25381330-25381311 (NC_010454.4)
*vWR*	gvWR F1/R1	Exon 2	Chromosome 5, 64553818-64553837 (NC_010447.5)
	gvWR F2/R2	Exon 3	Chromosome 5, 64556041-64556060 (NC_010447.5)
	gvWR F3/R3	Exon 4	Chromosome 5, 64557621-64557640 (NC_010447.5)
*ASGR1*	gASGR1 F1/R1	Exon 3	Chromosome 12, c52538530-52538511 (NC_010454.4)
	gASGR1 F2/R2	Exon 7	Chromosome 12, c52537633-52537614 (NC_010454.4)
	gASGR1 F3/R3	Exon 9	Chromosome 12, c52537146-52537127 (NC_010454.4)

^1^ Based on NCBI: Sus scrofa isolate TJ Tabasco breed Duroc chromosome 12, Sscrofa11.1, whole genome shotgun sequence, GCF_000003025.6.

**Table 2:** Comparison of the results of total efficiency predicted in silico with the results obtained in the in vitro cultured cells.

gRNA	In Silico Analysis Predicted Total Efficiency	In Vitro Analysis Total Efficiency
gGGTA1 F1/R1	78.9%	70.1% *
gGGTA1 F2/R2	72.2%	27.1%
gGGTA1 F3/R3	70.6%	18.2%
gCMAH F1/R1	61.5%	7.8%
gCMAH F2/R2	73.5%	12.9%
gCMAH F3/R3	70.1%	61.2% *
gβ4GalNT2 F1/R1	53.3%	8.1%
gβ4GalNT2 F2/R2	66%	17.6%
gβ4GalNT2 F3/R3	75.8%	45.2% *
gvWF F1/R1	48.9%	5.2%
gvWF F2/R2	73.9%	85.1% *
gvWF F3/R3	76%	39.6%
gASGR1 F1/R1	72.7%	13.9%
gASGR1 F2/R2	67.3%	33.5%
gASGR1 F3/R3	68.7%	80.5% *

* the gRNA together with total efficiency was marked, which was chosen as the best for disruption of the studied genes.

**Table 3:** A summary of bioinformatically predicted potential off-target sites for selected genetic constructs.

*Chosen* gRNA	*	Sequence	Porcine Genome Localization
gGGTA1 F1/R1	1.	G**C**TGC**A**C**T**TGAAGACCATCG	chr7: +33592796
	2.	GAT**AGT**CATG**G**AGACCATCG	chr7: +1925303 (*RIPK1* gene: ENSSSCG00000001009)
	3.	**CC**TGCGC**G**TGAAGACCA**A**CG	chr2: -44588168 (*OTOG* gene: ENSSSCG00000013376)
	4.	GA**G**G**T**GCATGAAGA**A**CATC**T**	chr2: +13174290
gCMAH F3/R3	5.	A**T**T**CG**ATCCTCCTAACCC**C**T	chr15: +40937188
	6.	**T**CT**T**AA**C**CCTC**A**TAACCCGT	chr4: -97128319
	7.	A**A**TAAATC**AC**CCTAACC**A**GT	chr4: +116709576 (*HIPK1* gene: ENSSSCG00000006760)
gβ4Gal NT2 F3/R3	8.	A**A**A**C**TACCA**G**CTCCACAGAG	chr16: -5677136
	9.	A**TT**GTACCACCTCCACAGA**C**	chr10: -13115375
	10.	**TC**AGTA**T**CACCTCCACAGAG	chr7: -109695811
gvWF F2/R2	11.	CC**T**T**C**T**G**CT**T**CATGCCCGCG	chr6: +157052374
	12.	**G**C**AC**GTACTCC**T**TGCCCGCG	chr4: -347951 (*ARHGAP39* gene: ENSSSCG00000005894)
	13.	CC**G**TGT**CG**TCCA**G**GCCCGCG	chr6: -9684887 (*WWOX* gene: ENSSSCG00000027415)
	14.	CCCTGT**C**CT**G**CA**G**GCC**T**GCG	chr14: -55036169 (*COMT* gene: ENSSSCG00000010132)
gASGR1 F3/R3	15.	**G**C**AT**ATG**T**CTGGTACGGGCA	chr6: +4310372
	16.	**G**C**AT**ATG**T**CTGGTACGGGCA	chr6: -4126764
	17.	C**ACA**ATGAC**A**GGTACGGGCA	chr5: +67768944 (*KCNA1* gene: ENSSSCG00000000716)
	18.	CC**A**GA**C**GACTGG**C**ACGGGCA	chr12: -54751930
	19.	CC**C**G**C**TG**T**CTGG**G**ACGGGCA	chr6: +155398812 (*C1orf210* gene: ENSSSCG00000003951)

Nucleotides not complementary to the target template for a given gRNA were determined with the bold font. * The number of the selected off-target site.

**Table 4:** Comparison of bioinformatic analysis with the results of laboratory analyses—analysis of the off-target sites presence.

gRNA	*	DNA Hydrolysis Efficiency at a Potential Off-Target Site	
		Bioinformatic Analysis	In Vitro Cultured Cells
**gGGTA1 F1/R1**	1.	1.6%	3.9%
	2.	0.5%	0%
	3.	0.4%	0%
	4.	0.2%	0.8%
**gCMAH F3/R3**	5.	0.6%	1.1%
	6.	0.6%	0%
	7.	0.2%	0%
**gB4GalNT2 F3/R3**	8.	2.6%	0%
	9.	1.8%	0%
	10.	1.8%	2.2%
**gvWF F2/R2**	11.	0.9%	1.8%
	12.	0.8%	0%
	13.	0.4%	0%
	14.	0.2%	3.4%
**gASGR1 F3/R3**	15.	1.5%	0%
	16.	1.5%	0%
	17.	1.4%	0%
	18.	0.7%	1.7%
	19.	0.6%	1.6%

* The number of the selected off-target site.

## Supplementary Materials

The indel spectrum results, insertion nucleotides probability results, alignment and decomposition quality controls for studied genetic constructs are available online at https://www.mdpi.com/2073-4425/11/6/713/s1, Figure S1: The indel spectrum and inserted nucleotide probability results, alignment, and decomposition quality controls for the other CRISPR/Cas9 genetic constructs containing gRNA tested for disruption of the porcine genes.; Figure S2: The alignment and decomposition quality controls for the CRISPR/Cas9 genetic constructs containing gRNA chosen as the best for the disruption of the porcine genes; Figure S3: The indel spectrum for the numbers 2–4 off-target loci after the use of CRISPR/Cas9 genetic construct containing gGGTA1 F1/R1 chosen as the best for disruption of porcine *GGTA1* gene; Figure S4: The indel spectrum for the numbers 5–7 off-target loci after the use of CRISPR/Cas9 genetic construct containing gCMAH F3/R3 chosen as the best for disruption of porcine *CMAH* gene; Figure S5: The indel spectrum for the number 8–10 off-target loci after the use of CRISPR/Cas9 genetic construct containing gβ4GalNT2 F3/R3 chosen as the best for disruption of porcine *β4GalNT2* gene; Figure S6: The indel spectrum for the numbers 11–14 off-target loci after the use of CRISPR/Cas9 genetic construct containing gvWF F2/R2 chosen as the best for disruption of porcine *vWF* gene; Figure S7: The indel spectrum for the numbers 15–19 off-target loci after the use of CRISPR/Cas9 genetic construct containing gASGR1 F3/R3 chosen as the best for disruption of porcine *ASGR1* gene.
