# Supplementary material for: Evaluation of the CRISPR/Cas9 Genetic Constructs in Efficient Disruption of Porcine Genes for Xenotransplantation Purposes Along with an Assessment of the Off-Target Mutation Formation
Source: Genes (Basel). 2020 Jun 26;11(6):713. doi: 10.3390/genes11060713 (PMC7349392; doi:10.3390/genes11060713)
Supplement: Supplementary file 1 [file genes-11-00713-s001.pdf]

Suplementary Data:

gGGTA1 F2/R2

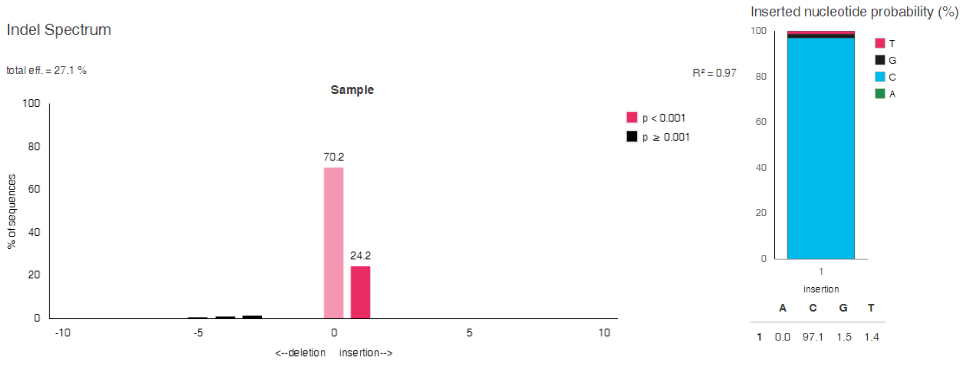

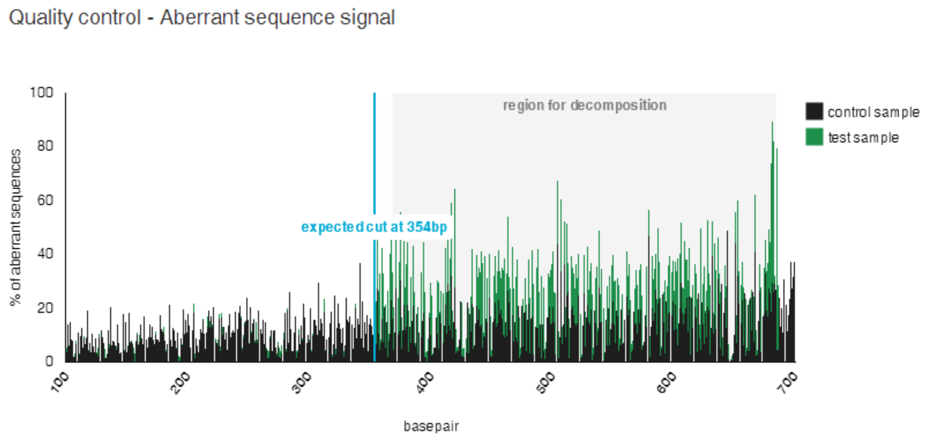

Alignment - local pairwise alignment score: 233.0

```
ctrl align: -------------ATAGAGCTGGGTCCTCTGCGTTCCTTTAAAGTGTTTGAGATCAAGTCCGAGAAGAGGTGGCAAGACATCAGCATGATGCGCATGAAGACCATC
GGGGAGCACATCCTGGCCCACATCCAGCACGAGGTGGACTTCCTCTTCTGCATGGACGTGGATCAGGTCTTCCAAAACAACTTTGGGGTGGAGACCCTGGGCCAGTCGGTGGCTCAGC
TACAGGCCTGGTGGTACAAGGCACATCCTGACNAG

edit align:  CNGGATGCCTTTGATAGAGCTGGGTCCTCTGCGTTCCTTTAAAGTGTTTGAGATCAAGTCCGAGAAGAGGTGGCAAGACATCAGCATGATGCGCATGAAGACCAT
CGGGGAGCACATCCTGGCCCACATCCAGCACGAGGTGGACTTCCTCTTCTGCATGGACGTGGATCAGGTCTTCCAAAACAACTTTGGGGTGGAGACCCTGGGCCAGTCGGTGGCTCAG
CTACAGGCCTGGTGGTACAAGGC-------------
```

(**a**)

gGGTA1 F3/R3

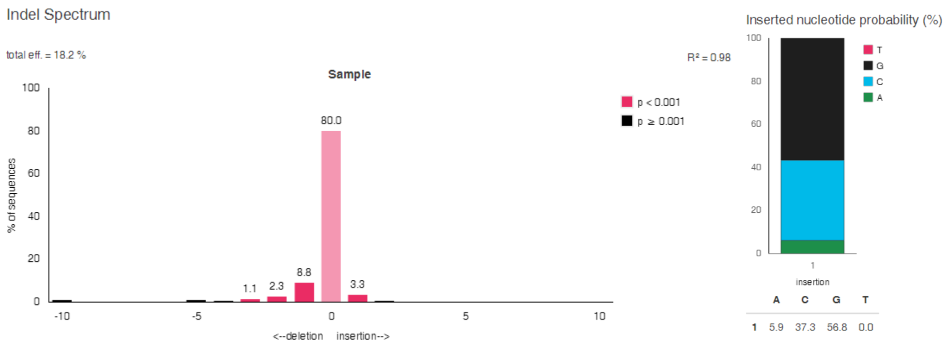

Quality control - Aberrant sequence signal

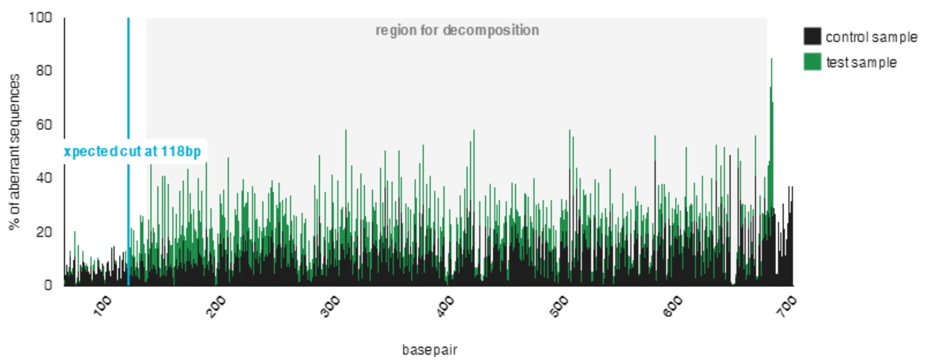

Alignment - local pairwise alignment score: 33.0

```
ctrl align: ----------------TTTTACATCATGGTGGATGATATCTCCAGGATGCCTTTGATAGAGCTGG

edit align:  TGGCCACAAAGTCATCTTTTACATCATGGTGGATGATATCTCCAGGATG----------------
```

(**b**)

gCMAH F1/R1

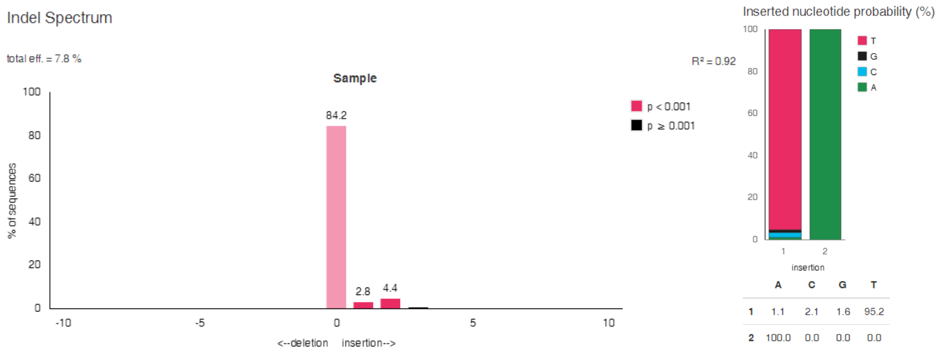

Quality control - Aberrant sequence signal

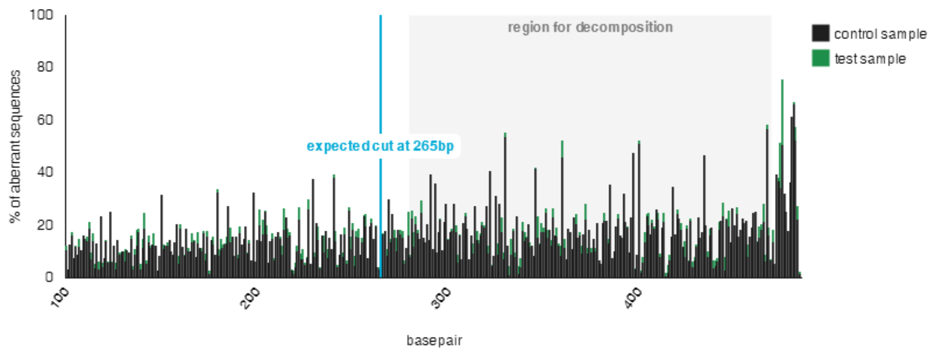

Alignment - local pairwise alignment score: 151.0

```
  ctrl align: -GGCTTGTCTGCTAACCAGCCCAGTTCCTTNTTCTGGCTGGTAATGATCCGATCATCTGAATCTCACTGTCTTCCAACAGATCACGTACCTTACTCACGCCNGC
ATGGACCTCAAGCTGGGNNACAAGAGNATGGTGTTCGANCCTTGGTTAATCG

  edit align:  TGGCTTGTCTGCTAACCAGCCCAGTTCCTTNTTCTGGCTGGTAATGATCCGATCATCTGAATCTCACTGTCTTCCAACANATCACGTACCTTACTCACGCCTG
CATGGACCTCAAGCTGGGNNACAANAGNATGGTGTTCGANCCTTGGTTAATC-
```

(**c**)

gCMAH F2/R2

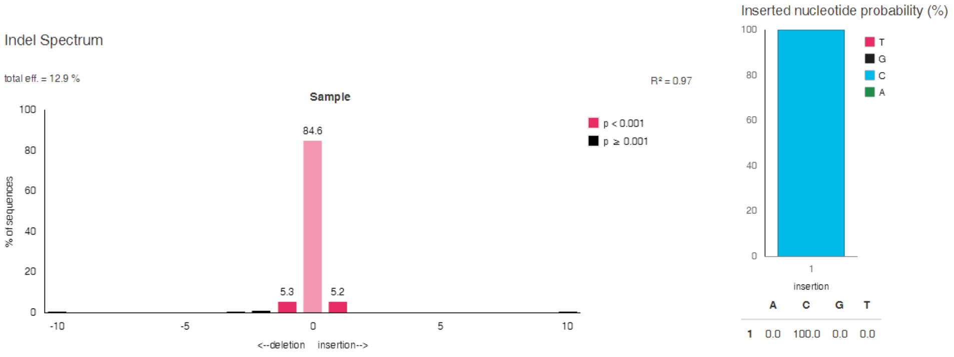

Quality control - Aberrant sequence signal

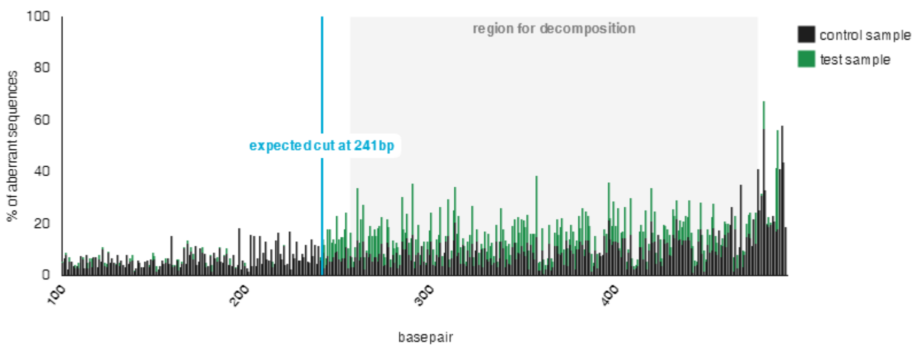

Alignment - local pairwise alignment score: 132.0

```
  ctrl align: -TCCTGTTGTGCCTCTCACCTGCCGAAGCTGCCAATCTCAAGGAAGGAATCAATTTTGTTCGAAATAAGAGCACTGGCAAGGATTACATCTTATTTAAGAATAA
GAGCCGCCTGAAGGCATGTAAGAACATGTG

  edit align:  ATCCTGTTGTGCCTCTCACCTGCCGAAGCTGCCAATCTCAAGGAAGGAATCAATTTTGTTCGAAATAAGAGCACTGGCAAGGATTACATCTTATTTAAGAATA
AGAGCCGCCTGAAGGCATGTAAGAACATGT-
```

(**d**)

gβ4GalNT2 F1/R1

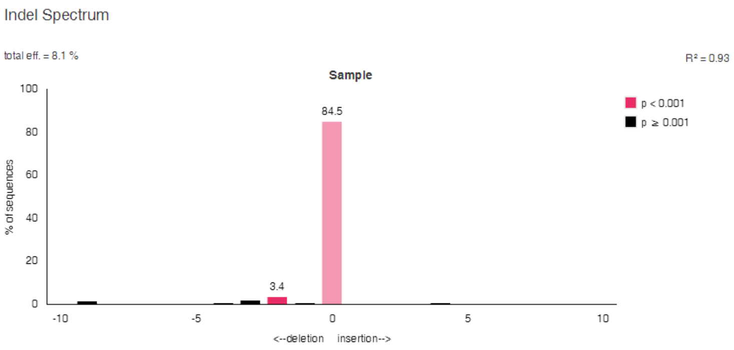

Quality control - Aberrant sequence signal

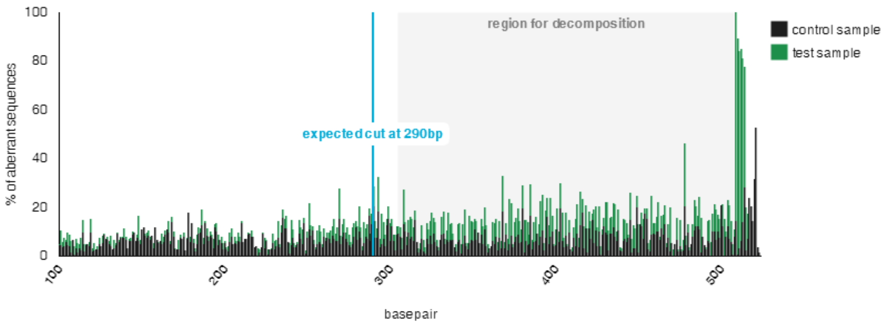

Alignment - local pairwise alignment score: 209.0

```
  ctrl align: --ATCAATTTTGATACGTAGTGTACNAAACAGGTTTGACTTGCATTTTTGCTCAAGTTGCTATTCCCATCTATGTCGCACAATGAAATCTTTTTTAACATACTC
AGAAAAGATTTTAACAGCGTTTGCTCTCTTGTGTCCCAACAGCCCTAGATGTCTGTCGATCCTCAAGATATNGATGGTGCTTTTGGTCCTGAGCGTTGGACTCTTTATG

  edit align:  AAATCAATTTTGATACGTAGTGTACNAAACAGGTTTGACTTGCATTTTTGCTCAAGTTGCTATTCCCATCTATGTCGCACAATGAAATCTTTTTTAACATACT
CAGAAAAGATTTTAACAGCGTTTGCTCTCTTGTGTCCCAACAGCCCTAGATGTCTGTCGATCCTCAAGATATNGATGGTGCTTTTGGTCCTGAGCGTTGGACTCTTTA--
```

(**e**)

gβ4GalNT2 F2/R2

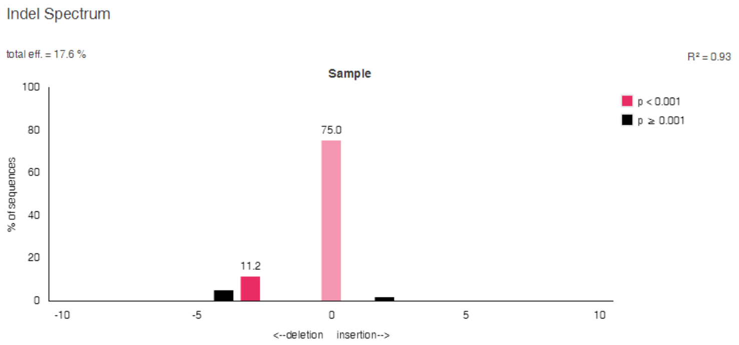

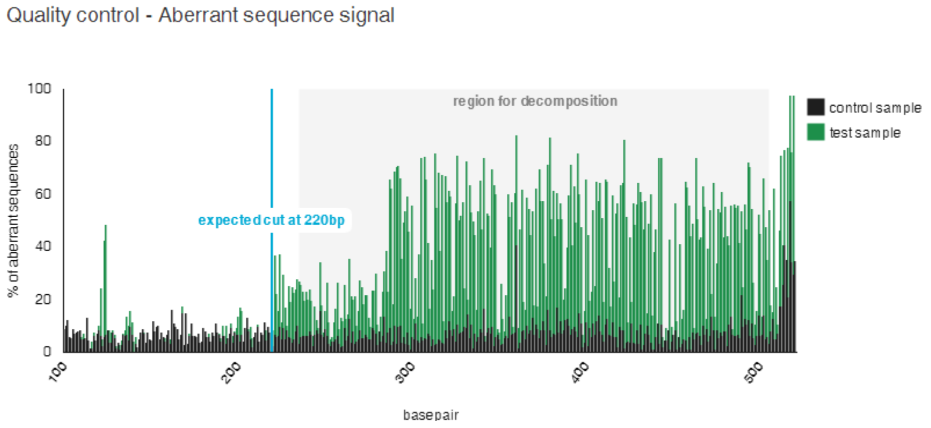

Alignment - local pairwise alignment score: 111.0

```
  ctrl align: CACGGATCTACTGAGTTTTACAGTCATCCCATTTGTAGACTTTTGCTACACCACCAAAGTATAGCATCTGAGATTAAATATTAATCTCCAAACCTTAGGCCCCC
TCACTTGC-

  edit align:  -ACGGATCTACTGAGTTTTACAGTCATCCCATTTGTAGACTTTTGCTACACCACCAAAGTATAGCATCTGAGATTAAATATTAATCTCCAAACCTTAGGCCCC
CTCACTTGCA
```

(**f**)

gvWF F1/R1

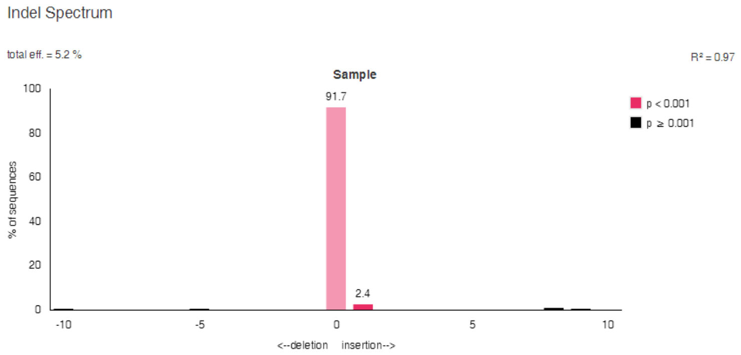

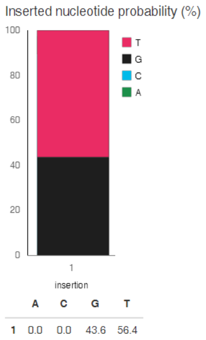

Quality control - Aberrant sequence signal

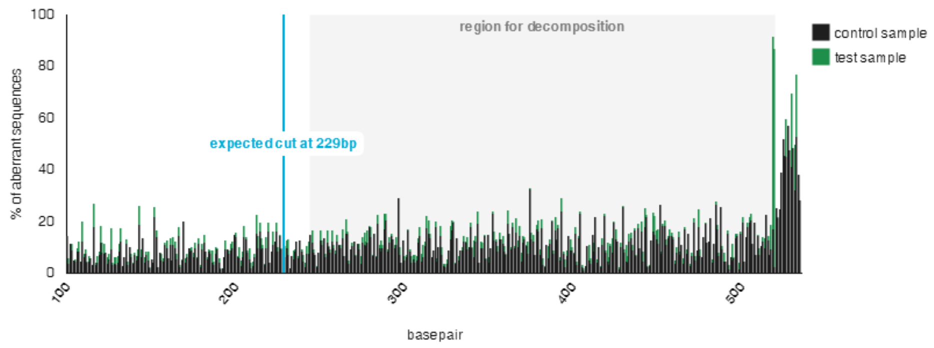

Alignment - local pairwise alignment score: 120.0

```
  ctrl align: -AGAGTGCCTCGGTGTTCGCCCGCTGCCACCCCCTGGTGGACCCCGAGCCTTTCGTGGCCCTGTGTGAGAAGATGCTGTGTCCATGTGCCCAGGGGCTGCAGTG
CCCGTGCCCCGCGCTCCT

  edit align:  AAGAGTGCCTCGGTGTTCGCCCGCTGCCACCCCCTGGTGGACCCCGAGCCTTTCGTGGCCCTGTGTGAGAAGATGCTGTGTCCATGTGCCCAGGGGCTGCAGT
GCCCGTGCCCCGCGCTCC-
```

(**g**)

gvWF F3/R3

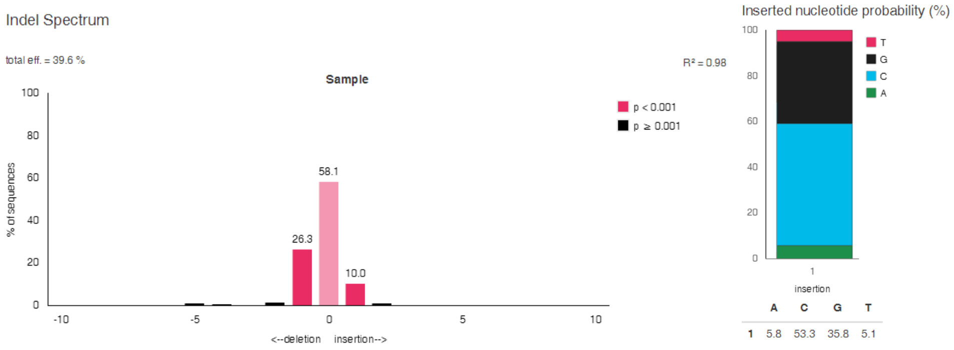

Quality control - Aberrant sequence signal

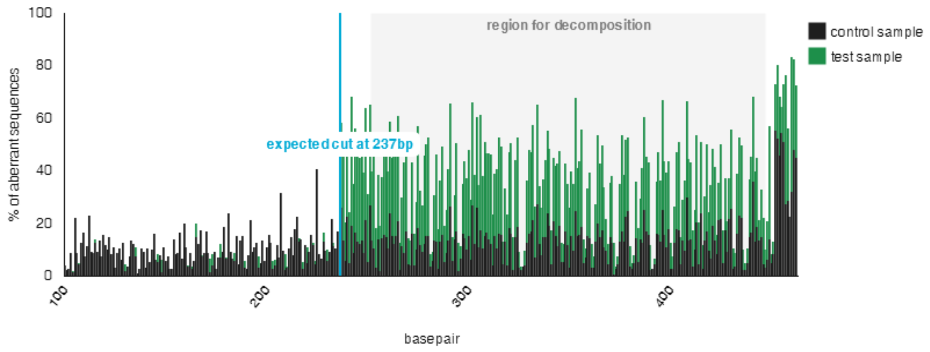

Alignment- local pairwise alignment score: 123.0

```
  ctrl align: CTTTTCGCAAGACGTGCTGAGCTGATGGTCACCCACGGGGATTCTGAGGTTATGGGATGAGTAAGGCTGATGACAAGGTTGAGTCTTGGCTTTGATGCCCTTGA
CCCANAGGGACAGCTCCTTGACNAC----

  edit align:  ----TCGCAAGACGTGCTGAGCTGATGGTCACCCACGGGGATTCTGAGGTTATGGGATGAGTAAGGCTGATGACAAGGTTGAGTCTTGGCTTTGATGCCCTTG
ACCCAGAGGGACAGCTCCTTGACGACGGCC
```

(**h**)

gASGR1 F1/R1

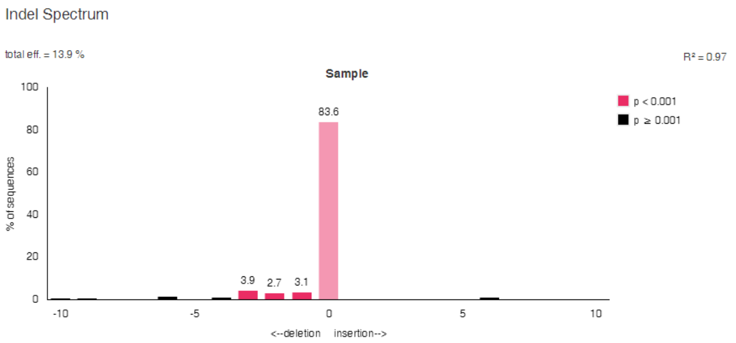

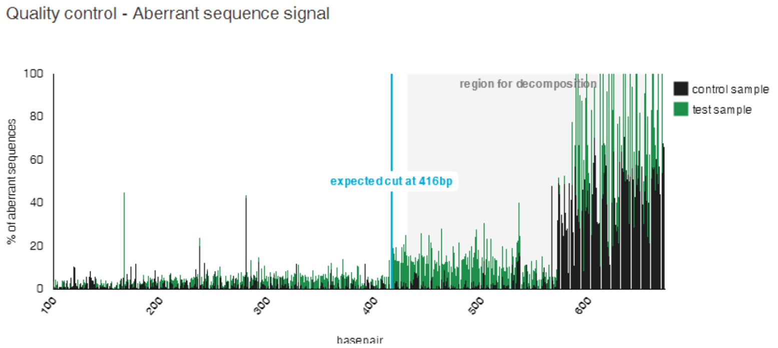

Alignment - local pairwise alignment score: 306.0

```
  ctrl align: --GTGTCTTTTCCTTCTTCTTTCCTTCCTTTCCCTTCCTTCCTCCCTCCCTTCCTTCTTTTTTGGCTGTGTCTACATGCAGAAATTCTTGGGCTAGGGATTGAA
CCCTTGCCATGGCAATGCTGGCTCTTTAACCCACCGAGCCACCAGGGAACTCCCATGTTTCTTAATCTATAAGGTTCCTGCTCGTCTACAAGAGGAGGCCCAGGATGCTCTCCTCCTA
GGACCCCAGAGCTGCCCTGCCCCAGGTCCCCTCTGTCCCTGAGGGCGTGACGCCCTCTCCTCTTGCAGGGCCACCTCCTCAACAGTCA

  edit align:  TCGTGTCTTTTCCTTCTTCTTTCCTTCCTTTCCCTTCCTTCCTCCCTCCCTTCCTTCTTTTTTGGCTGTGTCTACATGCAGAAATTCTTGGGCTAGGGATTGA
ACCCTTGCCATGGCAATGCTGGCTCTTTAACCCACCGAGCCACCAGGGAACTCCCATGTTTCTTAATCTATAAGGTTCCTGCTCGTCTACAAGAGGAGGCCCAGGATGCTCTCCTCCT
AGGACCCCAGAGCTGCCCTGCCCCAGGTCCCCTCTGTCCCTGAGGGCGTGACGCCCTCTCCTCTTGCAGGGCCACCTCCTCAACAGT--
```

(i)

gASGR1 F2/R2

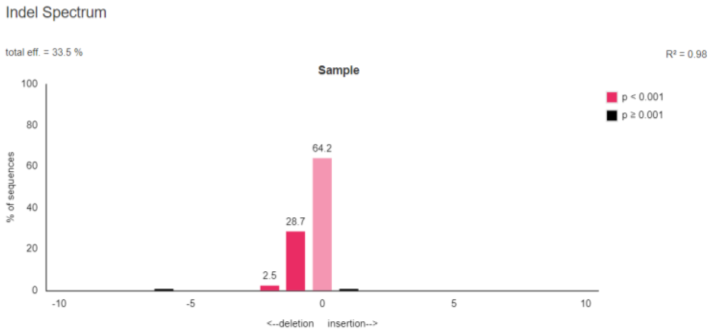

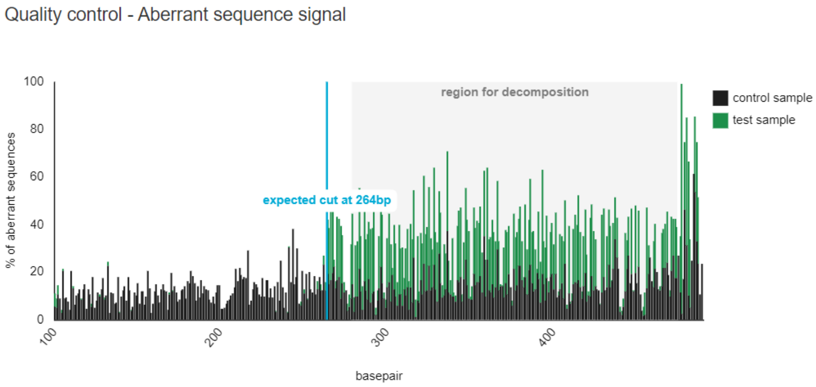

Alignment - local pairwise alignment score: 154.0

```
  ctrl align: --TGCGGCGCCCCGTGGCTCTGCCCCTCCAACTGCGCCCCCTCCCCAGTGGGCGGGTCCAGGGGTGCCCAGGCTGCTCCCCTCGGCTGAGGCTCTGGCCCCCTG
GGGCCCCCGTCCGTCTGGTCTCTTTAGGAACTGGAGACCCGANCAGCCGGATGA

  edit align:  TGTGCGGCGCCCCGTGGCTCTGCCCCTCCAACTGCGCCCCCTCCCCAGTGGGCGGGTCCAGGGGTGCCCAGGCTGCTCCCCTCGGCTGAGGCTCTGGCCCCCT
GGGGCCCCCGTCCGTCTGGTCTCTTTAGGAACTGGAGACCCGANCAGCCGGAT--
```

(j)

Figure S1. The indel spectrum and inserted nucleotide probability results, alignment and decomposition quality controls for the other CRISPR/Cas9 genetic constructs containing gRNA tested for disruption of the porcine genes. Results obtained after use the plasmids with: (**a**) gGGTA1 F2/R2; (**b**) gGGTA1 F3/R3; (**c**) gCMAH F1/R1; (**d**) gCMAH F2/R2; (**e**) gβ4GalNT2 F1/R1; (**f**) gβ4GalNT2 F2/R2; (**g**) gvWF F1/R1; (**h**) gvWF F3/R3; (**i**) gASGR1 F1/R1; (**j**) gASGR1 F2/R2.

gGGTA1 F1/R1

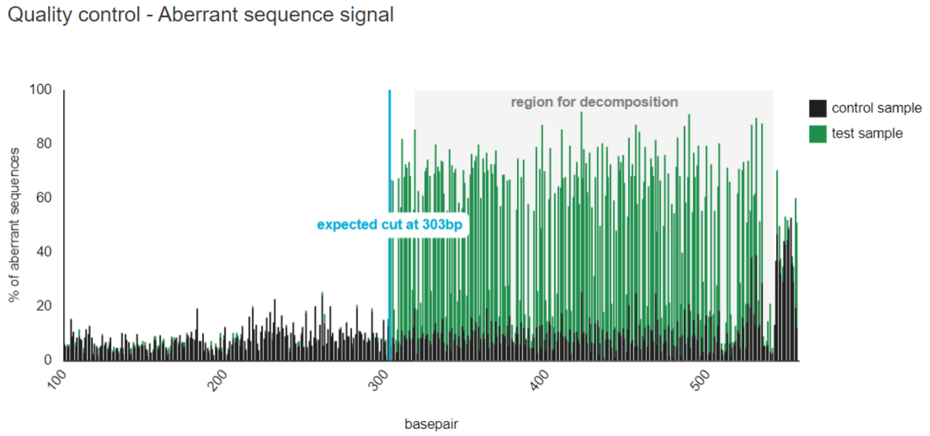

Alignment - local pairwise alignment score: 195.0

```
  ctrl align: GATACATTGAGCATTACTTGGAGGAGTTCTTAATATCTGCAAATACATACTTCATGGTTGGCCACAAAGTCATCTTTTACATCATGGTGGATGATATCTCCAGG
ATGCCTTTGATAGAGCTGGGTCCTCTGCGTTCCTTTAAAGTGTTTGAGATCAAGTCCGAGAAGAGGTGGCAAGACATCAGCATGATGCGCA

  edit align:  GATACATTGAGCATTACTTGGAGGAGTTCTTAATATCTGCAAATACATACTTCATGGTTGGCCACAAAGTCATCTTTTACATCATGGTGGATGATATCTCCAG
GATGCCTTTGATAGAGCTGGGTCCTCTGCGTTCCTTTAAAGTGTTTGAGATCAAGTCCGAGAAGAGGTGGCAAGACATCAGCATGATGCGCA
```

(**a**)

gCMAH F3/R3

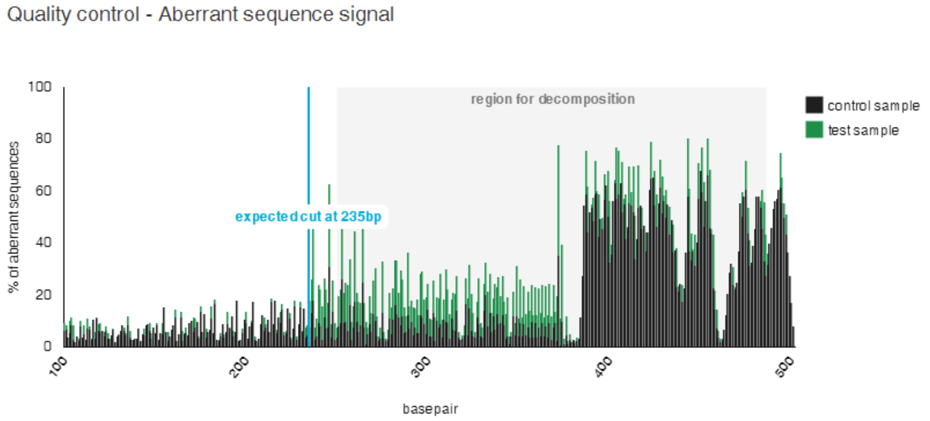

Alignment - local pairwise alignment score: 123.0

```
  ctrl align: ----CATGCATTTCTGTCTTAGCGATGCTTTTTAAAAGTCATTTTTTGGTTGATTATCCAGATTTGTCCACCTTTGCTTCTAGTTGTAGAAAAGGATGAAGAAA
ATGGAGTTTTGCTTCTAGAACTAAATC
```

```
  edit align:  GAGCCATGCATTTCTGTCTTAGCGATGCTTTTTAAAAGTCATTTTTTGGTTGATTATCCAGATTTGTCCACCTTTGCTTCTAGTTGTAGAAAAGGATGAAGAA
AATGGAGTTTTGCTTCTAGAACTA----
```

(**b**)

gβ4GalNT2 F3/R3

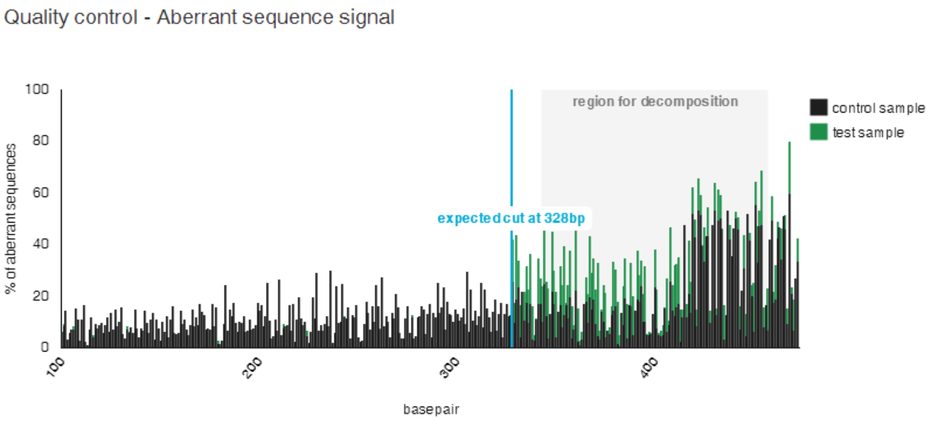

Alignment - local pairwise alignment score: 212.0

```
  ctrl align: TGCTGGCCGTCCTGGGTATGTGCCTCTCTGCATTTAGGCTTTCTACCTCCTTTATCCTAGGTCACCCTGACAGCTTCTCTGGGGACACTGAACACCCTTGCTGA
CGTCCCAGACAATGTGGTGAAGGGCANAGGCCAGAAGCAGCTGAACATTTTGACCAGTAGCCGGGAGCTTTTGAATTTCATCCTCCAGCATGTGACATACACGAGCACAGAGTACC--
------

  edit align:  --------GTCCTGGGTATGTGCCTCTCTGCATTTAGGCTTTCTACCTCCTTTATCCTAGGTCACCCTGACAGCTTCTCTGGGGACACTGAACACCCTTGCTG
ACGTCCCAGACAATGTGGTGAAGGGCANAGGCCAGAAGCAGCTGAACATTTTGACCAGTAGCCGGGAGCTTTTGAATTTCATCCTCCAGCATGTGACATACACGAGCACAGAGTACCA
CCTCCAC
```

(**c**)

gvWF F2/R2

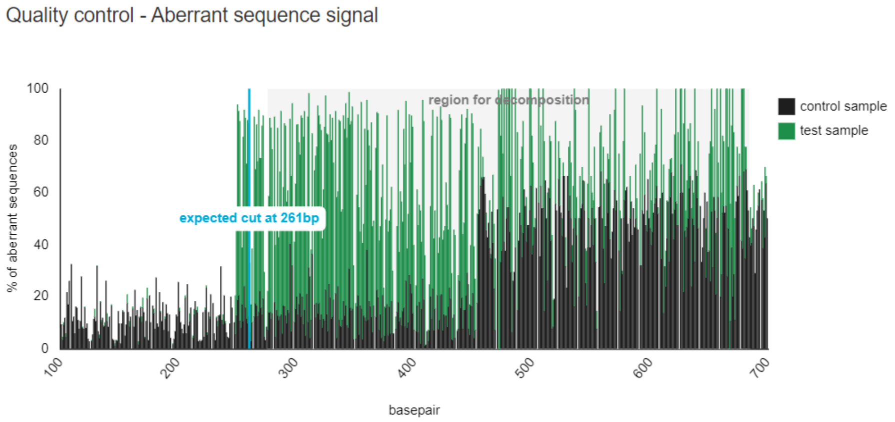

Alignment - local pairwise alignment score: 150.0

```
  ctrl align: -TGGCCTCTGGGAACCTGAAGATGAGGGGGGCAGACACTCAGCGGTGGGGGGAGGCAGGCTCTGAGCACCACATGGCTTGTGAGATGCTGCCTGAAGGGGGCGC
TGGGGAGACTCCTGATTTTTCCATTGCAGGGCCANACTGCCCCGCGGGCA

  edit align:  ATGGCCTCTGGGAACCTGAAGATGAGGGGGGCAGACACTCAGCGGTGGGGGGAGGCAGGCTCTGAGCACCACATGGCTTGTGAGATGCTGCCTGAAGGGGGCG
CTGGGGAGACTCCTGATTTTTCCATTGCAGGGCCAGACTGCCCCGCGGGN-
```

(**d**)

gASGR1 F3/R3

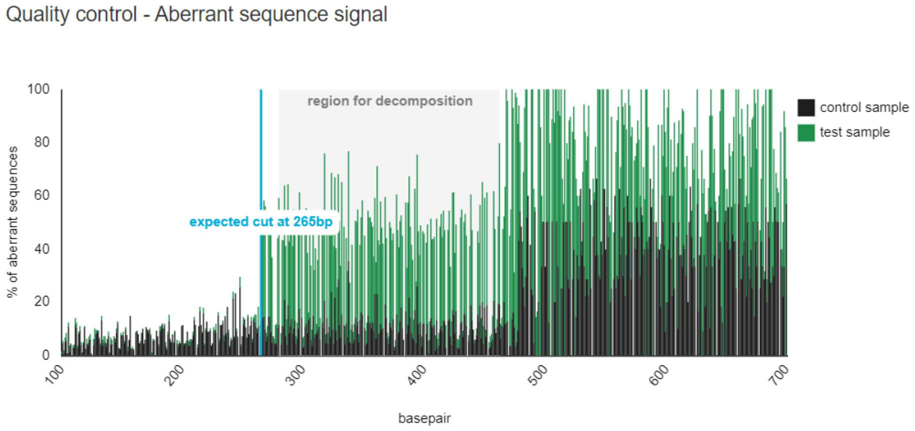

Alignment - local pairwise alignment score: 155.0

```
  ctrl align: GTGCGGCGCCCCGTGGCTCTGCCCCTCCAACTGCGCCCCCTCCCCAGTGGGCGGGTCCAGGGGTGCCCAGGCTGCTCCCCTCGGCTGAGGCTCTGGCCCCCTGG
GGCCCCCGTCCGTCTGGTCTCTTTAGGAACTGGAGACCCGAGCAGCCGGATGA--

  edit align:  --GCGGCGCCCCGTGGCTCTGCCCCTCCAACTGCGCCCCCTCCCCAGTGGGCGGGTCCAGGGGTGCCCAGGCTGCTCCCCTCGGCTGAGGCTCTGGCCCCCTG
GGGCCCCCGTCCGTCTGGTCTCTTTAGGAACTGGAGACCCGAGCAGCCGGATGACT
```

(**e**)

Figure S2. The alignment and decomposition quality controls for the CRISPR/Cas9 genetic constructs containing gRNA chosen as the best for the disruption of the porcine genes. Results obtained after use the plasmids with: (**a**) gGGTA1 F1/R1; (**b**) gCMAH F3/R3; (**c**) gβ4GalNT2 F3/R3; (**d**) gvWF F2/R2; (**e**) gASGR1 F3/R3.

Detailed results of the insertions and deletions obtained at potential off-target sites. Figure S3 shows the results for the genetic construct containing gGGTA1 F1/R1 excluding site No. 1 are shown in Figure 3.

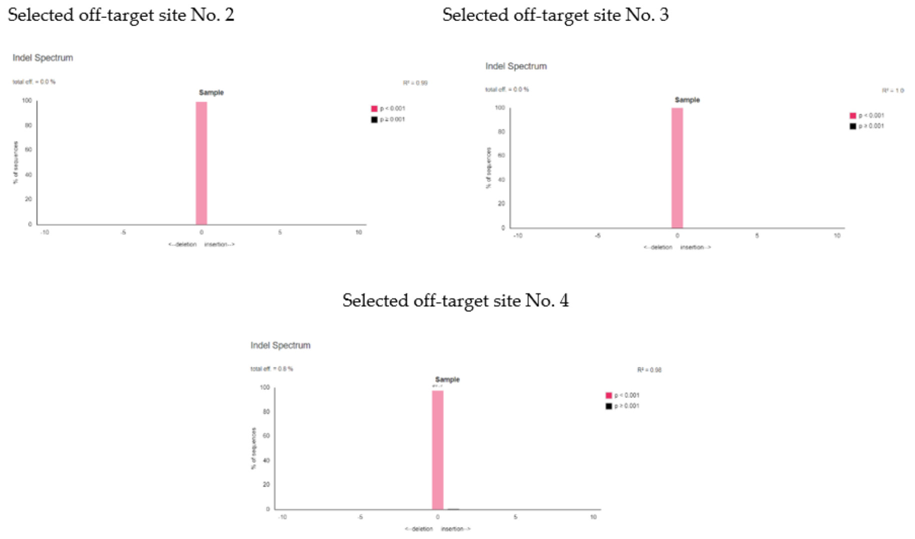

Figure S3. The indel spectrum for the numbers 2–4 off-target loci after the use of CRISPR/Cas9 genetic construct containing gGGTA1 F1/R1 chosen as the best for disruption of porcine *GGTA1* gene.

Figure S4 shows the indel spectrum results for the potential off-target sites for genetic construct containing gCMAH F3/R3.

Selected off-target site No. 5

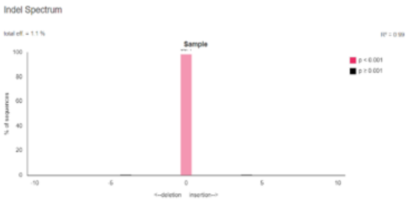

Selected off-target site No. 6

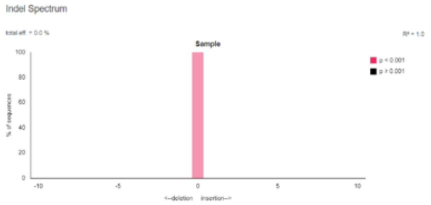

Selected off-target site No. 7

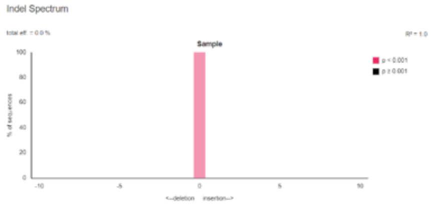

Figure S4. The indel spectrum for the numbers 5–7 off-target loci after the use of CRISPR/Cas9 genetic construct containing gCMAH F3/R3 chosen as the best for disruption of porcine *CMAH* gene.

Figure S5 shows the indel spectrum results for the potential off-target sites for genetic construct containing gβ4GalNT2 F3/R3.

Selected off-target site No. 8

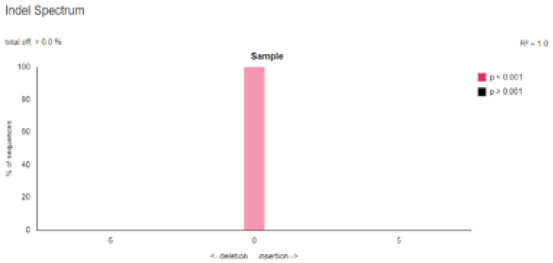

Selected off-target site No. 9

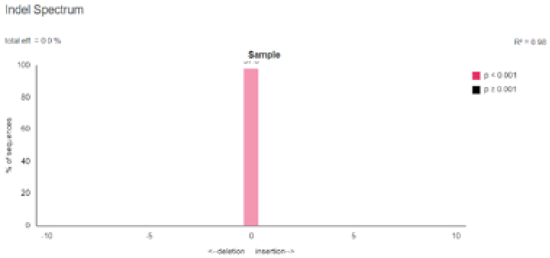

Selected off-target site No. 10

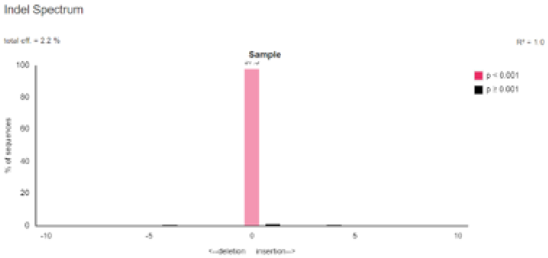

Figure S5. The indel spectrum for the number 8–10 off-target *loci* after the use of CRISPR/Cas9 genetic construct containing gβ4GalNT2 F3/R3 chosen as the best for disruption of porcine *β4GalNT2* gene.

Figure S6 shows the indel spectrum results for the potential off-target sites for genetic construct containing gvWF F2/R2.

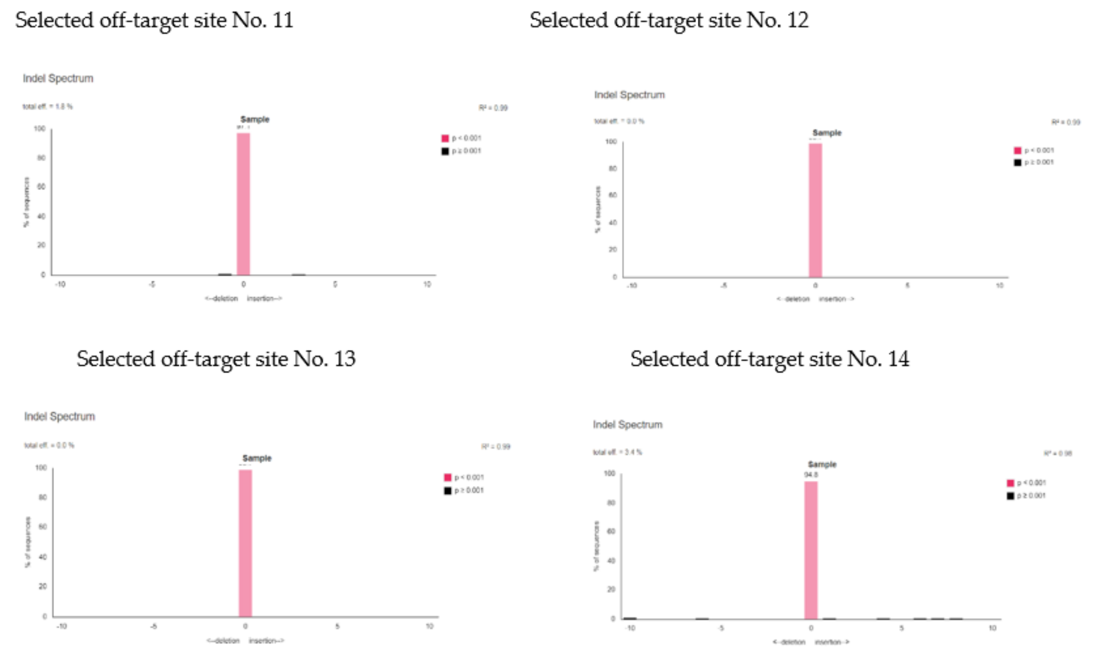

Figure S6. The indel spectrum for the numbers 11–14 off-target loci after the use of CRISPR/Cas9 genetic construct containing gvWF F2/R2 chosen as the best for disruption of porcine *vWF* gene.

Figure S7 shows the indel spectrum results for the potential off-target sites for genetic construct containing gASGR1 F3/R3.

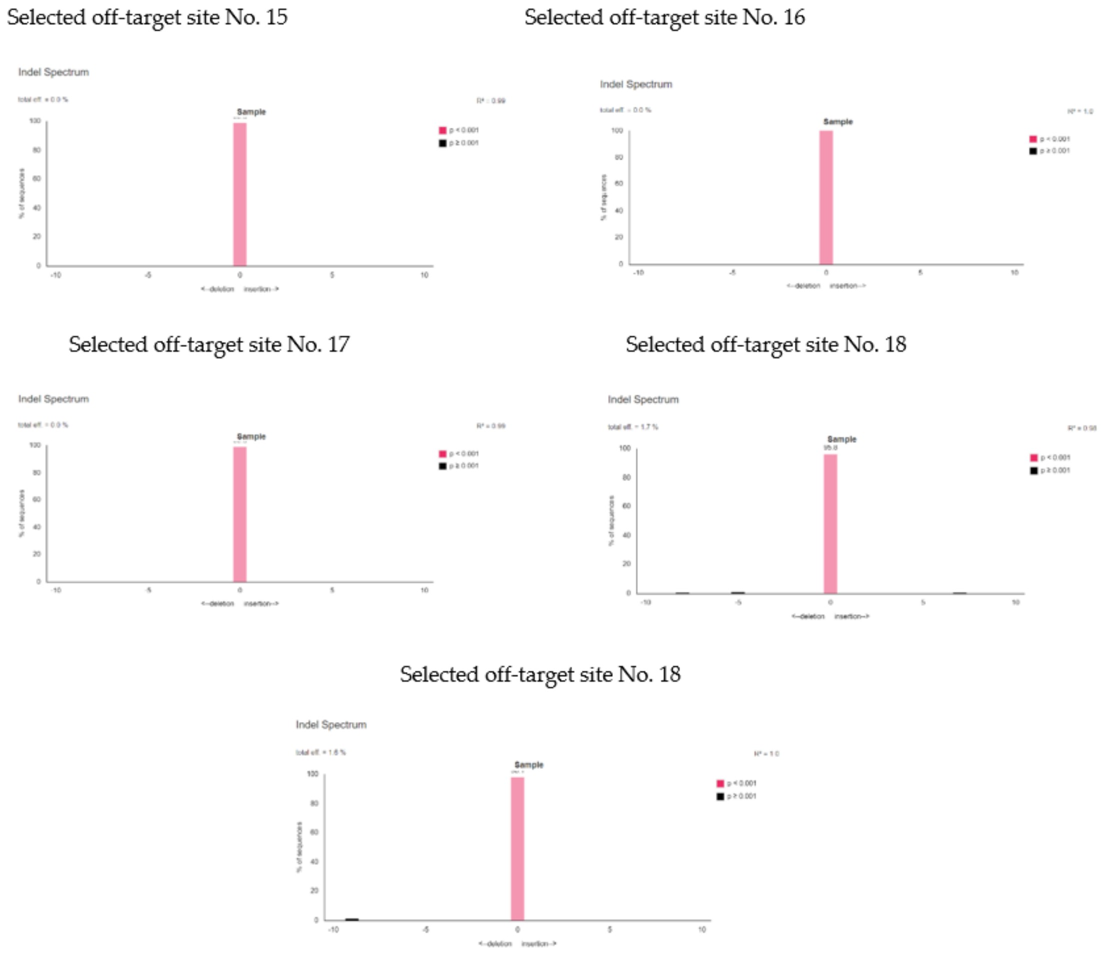

Figure S7. The indel spectrum for the numbers 15–19 off-target loci after the use of CRISPR/Cas9 genetic construct containing gASGR1 F3/R3 chosen as the best for disruption of porcine *ASGR1* gene.
